# Inhibition studies with simple and complex (in)organic anions of the γ-carbonic anhydrase from *Mammaliicoccus (Staphylococcus) sciuri,* MscCAγ

**DOI:** 10.1080/14756366.2023.2173748

**Published:** 2023-01-31

**Authors:** Simone Giovannuzzi, Viviana De Luca, Clemente Capasso, Claudiu T. Supuran

**Affiliations:** a Dipartimento Neurofarba, Sezione di Scienze Farmaceutiche e Nutraceutiche, Università degli Studi di Firenze, Sesto Fiorentino (Florence), Italy; b Department of Biology, Agriculture and Food Sciences, Institute of Biosciences and Bioresources, Napoli, Italy

**Keywords:** *Staphylococcaceae*, carbonic anhydrase, inhibitor, anion, *Mammaliicoccus (Staphylococcus) sciuri*

## Abstract

The γ-carbonic anhydrase (CA, EC 4.2.1.1) from the pathogenic bacterium, *Mammaliicoccus (Staphylococcus) sciuri* (MscCAγ) was recently cloned and purified by our groups. Here we investigated inhibition of this enzyme with (in)organic simple and complex anions, in the search of inhibitors with potential applications. The most effective inhibitors (K_I_s in the micromolar range) were peroxydisulfate and trithiocarbonate, whereas submillimolar inhibition was observed with *N,N*-diethyldithiocarbamate and phenylboronic acid (K_I_s of 0.5–0.9 mM). Thiocyanate, hydrogensulfide, bisulphite, stannate, divanadate, tetraborate, perrhenate, perruthenate, hexafluorophosphate, triflate and iminodisulfonate showed K_I_s of 1.0–13.7 mM. Cyanate, cyanide, azide, carbonate, nitrate, tellurate, selenocyanide, tetrafluoroborate, sulfamide, sulphamic acid and phenylarsonic acid were weaker inhibitors, with K_I_s in the range of 25.2–95.5 mM, whereas halides, bicarbonate, nitrite, sulphate, perchlorate and fluorosulfonate did not show inhibitory action up until 100 mM concentrations in the assay system. Finding more effective MscCAγ inhibitors may be helpful to fight drug resistance to antibiotics.

## Introduction

Carbonic anhydrases (CAs, EC 4.2.1.1) are present in prokaryotes and eukaryotes, with a large number of genetic families encoding them^1–4^. By efficiently catalysing the hydration of CO_2_ to bicarbonate and protons, these enzymes participate in many crucial physiologic processes, such as pH regulation^1–4^, metabolism (as they are involved in several biosynthetic pathways)^5^, photosynthesis (in plants and cyanobacteria)^6^, electrolyte secretion (in vertebrates)^7^, respiration and CO_2_/bicarbonate homeostasis^8^, just to mention the essential ones. Bacteria encode for at least 4 CA classes (α-, β-, γ- and ι-CAs)^2^^,^^4^ and in many pathogenic ones (but also in fungi or protozoans) the CAs are also involved in virulence, acclimation in specific tissues/organs and pathogen survival/fitness in environments possessing highly variable pH, bicarbonate and CO_2_ levels^2^^,^^9^. Thus, recently, inhibition of bacterial CAs started to be considered as a valid approach for obtaining antibiotics with a novel mechanism of action^2^^,^^10^^,^^11^, and at least for *Helicobacter pylori*^12^*, Neisseria gonorrhoeae*^11^^,^^13^ and vancomycin-resistant *Enterococci*^14^, detailed *in vitro*, *in vivo* and animal model proof-of-principle studies provided the experimental evidence that the approach is feasible and provides a powerful antibacterial effect^2^^,^^10–14^. Up until now, most such studies have been performed with sulphonamide CA inhibitors (CAIs) targeting these bacterial enzymes^10–14^. There are, however, many other less investigated chemotypes, such as the (in)organic anions, many of which act as zinc binders, as well as compounds possessing different inhibition mechanisms (e.g. anchoring to the zinc-coordinated water, occlusion of the active site entrance or out of the active site binding) which should be taken into consideration^15^. Apart natural products, which offer interesting structures for designing structurally novel enzyme inhibitors, including CAIs^16^, the investigation of simple (in)organic anions (or similar small molecules) afforded the discovery of new CAI classes, such as mono- and dithiocarbamates^15^, selenols^17^, ninhydrins^18^ and many other chemotypes^15^. Many such new types of reported CAIs were among the derivatives showing relevant CA isoform-selective inhibition profiles^15^.

Recently, we reported^19^ the cloning, purification and initial characterisation of a γ-class CA from the Gram-positive bacterium *Mammaliicoccus sciuri* (previously known as *Staphylococcus sciuri*), MscCAγ, which is responsible of infections in humans and various other wild/domestic animals, but also of gene transfer phenomena to other bacteria, that confer drug resistance to multiple antibiotics^19^. We hypothesised^19^ that inhibition of this enzyme may lead to a novel strategy for limiting the spread of multidrug resistance and investigated a range of sulphonamides and sulfamates as MscCAγ inhibitors. Some sulphonamides were indeed rather effective inhibitors, but compounds with an optimal selectivity profile for inhibiting the bacterial over the human enzymes have not yet been detected so far. Thus, here we report the investigation of (in)organic anions and structurally related small molecules as MscCAγ inhibitors, in the search of compounds with interesting inhibition profiles.

## Materials and methods

### Reagents

Anions and small molecules were commercially available reagents of the highest available purity from Sigma-Aldrich (Milan, Italy). The purity of tested compounds was higher than 99%.

### CA activity and inhibition measurements

An Applied Photophysics stopped-flow instrument has been used for assaying the CA catalysed CO_2_ hydration activity^20^. Phenol red at a concentration of 0.2 mM was used as pH indicator, working at the absorbance maximum of 557 nm, with 10 mM TRIS (pH 8.3) as buffer, and in the presence of 10 mM NaClO_4_ for maintaining constant the ionic strength, following the initial rates of the CA-catalysed CO_2_ hydration reaction for a period of 10–100 s. The CO_2_ concentrations ranged from 1.7 to 17 mM for the determination of the kinetic parameters and inhibition constants. For each inhibitor, at least six traces of the initial 5–10% of the reaction have been used for determining the initial velocity. The uncatalyzed rates were determined in the same manner and subtracted from the total observed rates. Stock solutions of inhibitors (10–50 mM) were prepared in distilled-deionized water and dilutions up to 0.01 µM were done thereafter with the assay buffer. Inhibitor and enzyme solutions were preincubated together for 15 min at room temperature prior to assay, in order to allow for the formation of the enzyme-inhibitor complex. The inhibition constants were obtained by non-linear least-squares methods using PRISM 3 and the Cheng-Prusoff equation, whereas the kinetic parameters for the uninhibited enzymes from Lineweaver-Burk plots, as reported earlier^21–23^, and represent the mean from at least three different determinations. MscCAγ was obtained as reported earlier^19^ and its concentration in the assay system was of 12.6 nM.

## Discussion and conclusions

Anions, both inorganic and organic ones, represent a relevant class of CAIs, which in most cases act as zinc binders^15^. They coordinate to the metal ion from the enzyme active site, frequently as monodentate ligands, with the preservation of the tetrahedral geometry of the active site metal ion, although rarely, an addition to the coordination sphere was observed, which led to trigonal bipyramidal geometries (at least for the zinc ion present in many classes of CAs^1^^,^^15^) Thus, we included in our study a large number of inorganic and organic anions, as well as small molecules structurally related to them (Table 1), known to interact with metal ions from metalloenzyme active sites^1^^,^^2^^,^^15^.

**Table 1.** Inhibition of human isoform hCA II and the bacterial enzyme from *Mammaliicoccus sciuri* (MscCAγ) with (in)organic anions and small molecules, by a CO_2_ hydrase, stopped-flow assay^20^.

Anion/small molecule	K_I_ (mM)^a^	
	hCA II	McsCAγ
F^–^	>300	>100
Cl^–^	200	>100
Br^–^	63	>100
I^–^	26	>100
CNO^–^	0.03	37.0
SCN^–^	1.6	9.3
CN^–^	0.02	61.8
N_3_^–^	1.5	89.2
HCO_3_^–^	85	>100
CO_3_^2–^	73	82.5
NO_3_^–^	35	95.5
NO_2_^–^	63	>100
HS^–^	0.04	7.2
HSO_3_^–^	89	3.7
SO_4_^2–^	>200	>100
SnO_3_^2–^	0.83	6.0
SeO_4_^2–^	112	0.70
TeO_4_^2–^	0.92	60.5
P_2_O_7_^4–^	48.5	0.8
V_2_O_7_^4–^	0.57	2.7
B_4_O_7_^2–^	0.95	1.0
ReO_4_^–^	0.75	4.4
RuO_4_^–^	0.69	4.9
S_2_O_8_^2–^	0.084	0.061
SeCN^–^	0.086	32.5
CS_3_^2–^	0.0088	0.070
Et_2_NCS_2_^–^	3.1	0.50
ClO_4_^–^	>200	>100
BF_4_^–^	>200	65.6
FSO_3_^–^	0.46	>100
PF_6_^–^	> 100t	1.7
CF_3_SO_3_^–^	> 100t	5.5
NH(SO_3_)_2_^2–^	0.76	13.7
H_2_NSO_2_NH_2_	1.13	49.2
H_2_NSO_3_H	0.39	25.2
Ph-B(OH)_2_	23.1	0.90
Ph-AsO_3_H_2_	49.2	67.1

^a^ Mean from 3 different assays, by a stopped flow assay (errors were in the range of ± 5–10% of the reported values, data not shown).

Data of Table 1, in which MscCAγ and hCA II (the human isoform II) anion inhibition data are presented, show the following inhibition profile of the new enzyme investigated here (human enzyme data are presented for comparison reasons):

- The following anions did not inhibit MscCAγ up to 100 mM concentration in the assay system: all halides, bicarbonate, nitrite, sulphate, perchlorate and fluorosulfonate. The last anion is a potent hCA II inhibitor (Table 1) whereas the remaining ones also poorly inhibit the human isoform (apart iodide which is a medium potency hCA II inhibitor).
- Weak inhibition of MscCAγ has been observed with cyanate, cyanide, azide, carbonate, nitrate, tellurate, selenocyanide, tetrafluoroborate, sulfamide, sulphamic acid and phenylarsonic acid, which presented K_I_s in the range of 25.2–95.5 mM. Some of these anions/compounds (e.g. nitrate, tellurate, tetrafluoroborate) are known to possess low affinity to complexate metal ions in solution or within enzymes active sites^24^. However, cyanate, cyanide, azide, and other anions have a very high affinity for cations^24^, and it is thus surprising that so diverse anions possess similar inhibition constants for MscCAγ. The same should be mentioned on sulfamide and sulfanic acid (sulfamate), which effectively inhibit α-CAs (including hCA II, as shown in Table 1)^25^, whereas their activity on MscCAγ is quite weak.
- Effective MscCAγ inhibitory activity has been registered for thiocyanate, hydrogensulfide, bisulphite, stannate, divanadate, tetraborate, perrhenate, perruthenate, hexafluorophosphate, triflate and iminodisulfonate, which showed K_I_s in the range of 1.0–13.7 mM. Again, some of these anions (thiocyanate, hydrogensulfide) are very good metal complexing agents, whereas others (haxafluorophosphate, triflate, etc.) are well-known for their low affinity for metal ions^25^. Thus, it is difficult to rationalise these results, considering the fact that such effects have been described for other CAs earlier (e.g. for hCA II)^15^^,^^25^.
- Submillimolar MscCAγ inhibitory activity has been evidenced for selenite, pyrophosphate, *N,N*-diethyldithiocarbamate and phenylboronic acid (K_I_s in the range of 0.5–0.9 mM), whereas peroxydisulfate and trithiocarbonate were the most effective inhibitors, with inhibition constants of 60 and 70 µM, respectively.
- The inhibition profiles with (in)organic anions of the bacterial and human CAs investigated are very different, which signifies that it might be possible to design inhibitors selective for the pathogenic enzyme, as already demonstrated for other bacterial CAs^2^^,^^10–12^.

In conclusion, we report here the first inhibition study with (in)organic anions and small molecules (sulfamide, sulphamic acid, phenylboronic acid and phenylarsonic acid) of MscCAγ. The most effective inhibitors (K_I_s in the micromolar range) were peroxydisulfate and trithiocarbonate, whereas submillimolar inhibition was observed with *N,N*-diethyldithiocarbamate and phenylboronic acid (K_I_s of 0.5–0.9 mM). Thiocyanate, hydrogensulfide, bisulphite, stannate, divanadate, tetraborate, perrhenate, perruthenate, hexafluorophosphate, triflate and iminodisulfonate showed K_I_s of 1.0–13.7 mM. Cyanate, cyanide, azide, carbonate, nitrate, tellurate, selenocyanide, tetrafluoroborate, sulfamide, sulphamic acid and phenylarsonic acid were weaker inhibitors, with K_I_s in the range of 25.2–95.5 mM, whereas halides, bicarbonate, nitrite, sulphate, perchlorate and fluorosulfonate did not show inhibitory action up until 100 mM concentrations in the assay system. Although we did not find highly effective MscCAγ inhibitors among the small molecules and anions investigated here, some of the tested compounds, such as N,N-diethyldithiocarbamate, phenylboronic acid or trithiocarbonate, which showed relevant inhibition, may be developed and used as leads for the design of presumably more effective bacterial CA inhibitors.
